# Circumferential Bulging Variation and Temperature Distribution of Profile-Tunable Roll for Freeform Optics in Roll-to-Plate (R2P) Hot-Embossing Process

**DOI:** 10.3390/mi16121395

**Published:** 2025-12-11

**Authors:** Yanfeng Feng, Lixiong Luo, Yujie Zhou, Zhiqiang Xu, Tingsong Yang, Chao Hong, Benshuai Ruan, Shengwei Li, Chao Yan

**Affiliations:** 1 Shenzhen Key Laboratory of High-Performance Nontraditional Manufacturing, College of Mechatronics and Control Engineering, Shenzhen University, Shenzhen 518060, China; yyfeng@szu.edu.cn (Y.F.); 2500091006@mails.szu.edu.cn (L.L.); zhouyujie0118@163.com (Y.Z.); 2 Guangdong Provincial Key Laboratory of Micro/Nano Optomechatronics Engineering, College of Mechatronics and Control Engineering, Shenzhen University, Shenzhen 518060, China; 3 School of Mechanical Engineering, Yanshan University, Qinhuangdao 066004, China; xzq@ysu.edu.cn (Z.X.); tsyang324@163.com (T.Y.); 4 Beijing Yiyong Technology Co., Ltd., Beijing 101300, China; hongchao@cheerfulnano.com; 5 Chaofeng Micro-Nano Technology (Ningbo) Co., Ltd., Ningbo 315502, China; ruanbenshuai@cheerfulnano.com (B.R.); lishengwei@cheerfulnano.com (S.L.)

**Keywords:** roll-to-plate hot embossing, circumferential bulging variation, circumferential temperature distribution, profile-tunable roll, freeform optical plates

## Abstract

The roll-to-plate (R2P) hot-embossing process is a newly developed molding technique for the high-throughput, high-efficiency fabrication of large-area microstructured optical elements. However, this technology is limited to flat surfaces, because the thickness of the freeform optical plate varies constantly due to its specific optical design, while the roll stays cylindrical during rolling. Therefore, we developed a new profile-tunable roll with several groups of semiconductor heater/coolers (SHCs) attached around the inside wall of the roll. These SHCs can achieve tunable roll profiles at desirable positions by regulating the current for the semiconductor and then the roll temperature, thereby producing optics with a selected freeform. In this paper, the circumferential bulging profiles and corresponding roll temperature fields were thoroughly investigated under various heater/cooler layouts and roll sizes. A circumferential finite element model of the profile-tunable roll was established using the finite element software MSC.MARC 2020 and then verified on the experimental platform. In addition, the fundamental relationship between the bulging values and temperature distributions of the roll and parameters, such as the outer diameter and inner diameter of the roll, the temperature of the semiconductor heater/cooler, and the single piece influence angle, was eventually established. This paper offers a unique fabrication method for high-volume optical freeform plates at extremely low cost and builds a foundation for further research on the axial deformation and temperature distribution of the developed roll for freeform optics and R2P hot-embossing experiments for freeform optical components.

## 1. Introduction

Non-spherical elements, particularly freeform optics, can effectively mitigate optical aberrations and internal reflections, reduce the number of lenses, and consequently streamline assembly requirements in various applications [1,2,3]. Continuous advancements in research and development have rendered freeform optical surfaces increasingly viable for addressing diverse design and manufacturing challenges. Such asymmetrical surfaces encompass torics, biconics, and bi-aspherics; phase masks; f-θ lenses for scanners; progressive lenses for eyewear; lens arrays; and lenses requiring off-axis machining, such as conformal optics, among a myriad of other applications that are yet to be precisely defined.

Traditional methods for fabricating polymeric freeform optics have primarily involved microinjection [4] and microscale hot embossing [5]. Notably, Li et al. proposed a fabrication method using a combination of ultraprecision diamond machining and microinjection molding to achieve high-volume and low-cost freeform microlens manufacturing [6]. After that, their group created a freeform microlens array residing on a flat substrate with a large field of view of 48° × 48° [7]. Joo et al. presented a design procedure and compression molding process for LED primary optics as functional road lighting secondary optics [8].

However, traditional manufacturing methods suffer from drawbacks like low processing efficiency, a limited microstructure replication rate, and size constraints. Consequently, there is a pressing need for a breakthrough technology that enables the continuous production of large-area freeform optics, thereby enhancing production efficiency and reducing costs. Addressing this need, the development of R2P hot embossing provides a turnkey solution, which involves a roller mold and a plate mold [9,10,11]. The technique uses pressure and heat to stamp 3D microstructures from a roller or plate mold onto glass. Rather than the point-to-point contact of standard P2P embossing, R2P provides a pseudo-1D interface, enabling continuous, large-area patterning by translating the contact line across the substrate [12]. Following a series of studies, Zhu et al. demonstrated the effective replication of surface microstructures onto glass substrates through R2P hot embossing [13].

However, fabrication for large-area freeform optics can hardly be achieved by R2P hot-embossing technology unless the roll profiles can be regulated according to set parameters. Therefore, we creatively attempt to introduce roll profile dynamic control technology into the R2P hot-embossing process to tune the roll profiles to perfectly match the freeform surface of optical plates, thereby achieving efficient large-area freeform optics fabrication.

Until now, roll-profile dynamic control technologies for alloy strip rolling have only achieved real-time online adjustments of the roll profile by regulating internal mechanical or thermal loads. Representative approaches include variable crown rolls [14], dynamic shape rolls [15], thermally bulging rolls [16], and electromagnetic roll-profile control [17]. Compared with force-driven methods, heat-driven technologies provide a larger regulation range of the roll profile because thermal expansion and contraction under heating and cooling are more pronounced. This is particularly attractive for freeform optical surfaces, which typically require large-radius profiles with small curvatures.

Considering the thermal behavior of the rolls, Park et al. [18] identified heat flux, roll width, and rolling speed as the main factors governing the development of thermal convexity in twin-roll casting. Benasciutti et al. [19] showed that periodic heat sources generate nonuniform surface temperature distributions and, consequently, nonuniform thermal stresses. Jiang et al. [20] developed a precision online model of the thermal crown that accounts for the effects of external periodic cooling and rolling heat input. Chen et al. [21] established a finite-difference model for the online calculation of the roll temperature field and reported that the adjustable range of the thermal crown due to working-roll shifting is approximately ±30 μm.

These studies provided detailed insights into how the temperature field affects the thermal roll profile; however, the circumferential boundary conditions were simplified as axisymmetric to facilitate the analysis. As a result, previous work has mainly focused on axial heat-transfer characteristics, and circumferential heat transfer has received much less attention. With respect to circumferential heat transfer, Yang et al. [22] investigated the effect of circumferential slot structures in the electromagnetic stirrer on the bulging magnitude and uniformity in electromagnetic roll-profile control, and they found that increasing the number and size of slots reduces the bulging value. Hajmohammadi et al. [23] analyzed the heat-transfer performance of fin arrays in a circular channel and demonstrated that a nonuniform fin configuration can increase the heat-exchange capacity by 46% relative to a conventional smooth channel. Ding et al. [24] examined the heat-dissipation characteristics of vertically oriented three-dimensional (3D) finned tubes and showed that their Nusselt number is 207% higher than that of smooth tubes.

The above studies focus on the circumferential heat-transfer capacity of cylindrical structures, but their application to the roll-to-plate (R2P) hot-embossing process for optical plates has rarely been reported. Therefore, this study introduces a new profile-tunable roll into the R2P hot-embossing process to actively adjust the roll profile by controlling several groups of semiconductor heater/cooler (SHC) modules. These SHCs enable the rapid internal heating and cooling of the roll, thereby precisely regulating the internal temperature field and flexibly tuning the local thermal roll profile. By coordinating multiple SHC ring groups distributed along the roll axis, the overall roll shape can be tailored in both the radial and axial directions.

In this work, SHCs are integrated into the R2P hot-embossing process, and the circumferential bulging profiles and corresponding roll temperature fields under different heater/cooler layouts and roll sizes are systematically investigated. The relationships between the circumferential profiles and the SHC parameters and layouts are studied by establishing a finite element model (FEM) in MSC.MARC. After experimental validation, the model is used to predict the bulging amplitudes and radial temperature distributions of the roll. In addition, a copper layer is inserted inside the roll to tune the circumferential heat transfer, thereby enabling different temperature distributions and circumferential roll profiles.

## 2. Design of Profile-Tunable Roll

**Figure 1:**
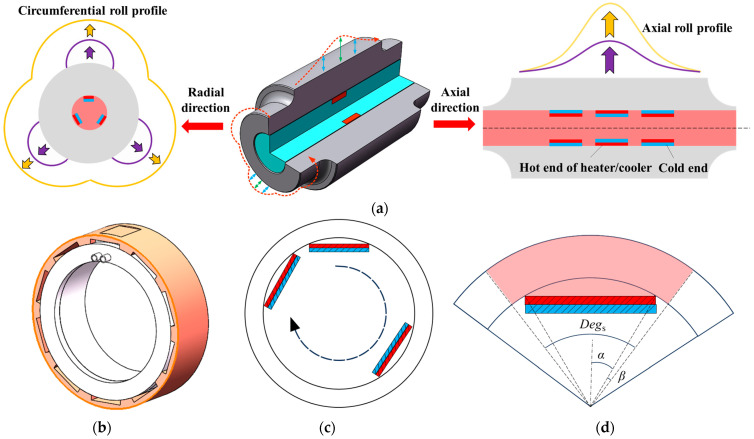
Schematic of (**a**) the profiles produced by profile-tunable roll in radial direction and axial direction, where purple and yellow arrows represent the bugling direction; (**b**) SHC ring group, (**c**) SHC layout in the circumferential direction; and (**d**) relationships between single-piece influence angle *Deg_s_*, direct influence angle *α*, and indirect influence angle *β* in SHC.

In the design of the proposed profile-tunable roll, multiple SHCs are arranged closely along the axial direction to form a constant and uniform profile in the axial direction while arranged separately in the circumferential direction to provide a curved and changing roll gap to form the freeform surfaces. As presented in Figure 1a, the profiles produced by SHC along the axial direction and radial direction can be clearly distinguished, and in this paper, we fix the profiles along the axial direction as constant and tune the profiles in the radial direction to achieve the shape we want. Each SHC ring group, as shown in Figure 1b, can offer an independent heat or cold source to the localized roll zone. In one SHC ring group, a plurality of SHCs are arranged circumferentially at the same equal angle to form a whole SHC ring group in Figure 1c, which is used to adjust the roll profile in this section. To investigate the effect of each SHC on the localized temperature field and roll profile, we define some angle variables to facilitate subsequent discussions. Figure 1d shows a sketch of the geometrical relationship between the single-piece influence angle *Deg_s_*, the direct influence angle *α*, and indirect influence angle *β*.

In the design of the proposed profile-tunable roll, multiple SHCs are closely arranged along the axial direction to form an axially constant and uniform roll profile, whereas they are discretely distributed in the circumferential direction to generate a curved and spatially varying roll gap for forming freeform surfaces. As illustrated in Figure 1a, the SHC-induced profiles in the axial and radial directions can be clearly distinguished. In this study, the axial profile is kept constant, and only the radial profile is tuned to obtain the desired roll shape. Each SHC ring group, as shown in Figure 1b, provides an independent heating or cooling source for a local roll zone. Within one SHC ring group, multiple SHCs are arranged circumferentially at equal angular intervals to form a complete SHC ring, as depicted in Figure 1c, which is used to adjust the roll profile in the corresponding axial section. To analyze the influence of each SHC on the local temperature field and roll profile, several angular variables are defined for subsequent discussion. Figure 1d shows a schematic of the geometric relationship among the single-piece influence angle *Deg_s_*, the direct influence angle *α*, and the indirect influence angle *β*.

The single-piece influence angle *Deg_s_* is the circumferential angle that can be affected by a single SHC when a plurality of SHCs are used to form a SHC ring group. Its calculation formula can be shown in Equation (1):

(1) $$Deg_{s}=\frac{2\pi }{n}$$

The direct influence angle *α* is the circumferential angle of the area adhered to by a single SHC. Its calculation formula can be shown in Equation (2):

(2) $$\alpha =\arcsin\frac{L}{2R}$$

where *R* is the roll radius of the profile-tunable roll, and *L* is the length of a single SHC.

The indirect influence angle *β* is the angle except for the area adhered to by a single SHC. Its calculation formula can be shown in Equation (3):

(3) $$\beta =\frac{\pi }{n}-\arcsin\frac{L}{2R}$$

If the SHC control temperature is set to *T_c_*, the circumferential function of the heat source in the roll inner wall can be obtained according to the periodic distribution characteristics of the SHC layout, as shown in Equation (4):

(4) $$f\left(\theta \right)=\left\{\begin{array}{ll} T_{c} & \frac{2\pi }{n}\left(i-1\right)-\alpha <\theta <\frac{2\pi }{n}\left(i-1\right)+\alpha \\ 0 & \frac{2\pi }{n}\left(2i-3\right)\leq \theta \leq \frac{2\pi }{n}\left(i-1\right)-\alpha \cup \frac{2\pi }{n}\left(i-1\right)+\alpha \leq \theta \leq \frac{2\pi }{n}\left(2i-1\right) \end{array}\right.$$

where *i* = 1, 2, 3, …, *n*.

**Figure 2:**
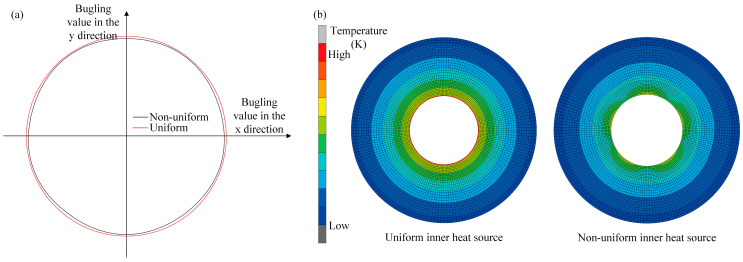
Difference of roll bulging ability and heat source level under (**a**) uniform and (**b**) non-uniform distribution of SHC heat source.

Because the SHC heat sources are non-uniformly distributed along the inner hole of the profile-tunable roll, freeform optical plates can be formed during the R2P hot-embossing process. Figure 2 compares the roll bulging response and heat-source intensity for uniform and non-uniform SHC distributions. The analysis in Figure 2 indicates that when the equivalent uniform temperature is higher than the nominal SHC-ring temperature, the actual expansion capability of the profile-tunable roll cannot be evaluated accurately. Bulging occurs only when the temperature field is circumferentially non-uniform; in this case, the resulting non-uniform thermal expansion generates the desired bulging profile. Therefore, it is necessary to analyze the coupled effects of the temperature distribution and roll profile to determine the applicability of the equivalent-temperature assumption.

## 3. Circumferential FE Simulation of Profile-Tunable Roll

**Figure 3:**
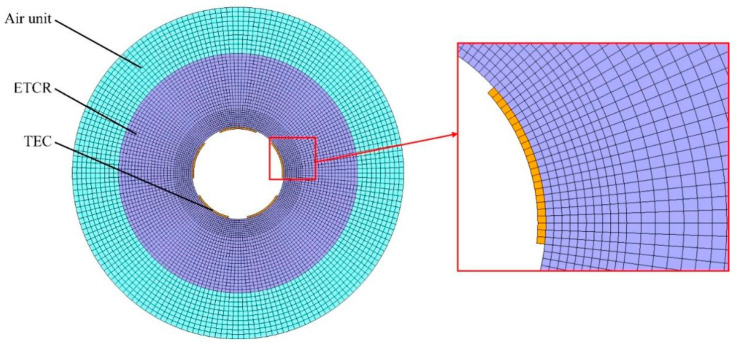
Circumferential FE model of profile-tunable roll.

Figure 3 shows the circumferential finite element (FE) model of the profile-tunable roll, which is used to analyze the non-uniform temperature field in the circumferential direction. Because the SHC modules are typically closely packed along the axial direction, the model can be simplified to a plane-strain formulation. A circumferential slice is modeled in plane strain to capture the azimuthal behavior directly driven by the SHCs. For the hardware considered, the roll length is large compared with the circumferential wavelengths of interest, and the modules are uniformly arranged along the axis. Consequently, the axial distribution is expected to have a similar shape and mainly modulate the extrema rather than the circumferential pattern; axial end effects and localized cooling hardware are therefore outside the scope of this study. The model includes an SHC heat source, silicone grease, an electronic temperature control roll, and an air unit. According to the principle of profile-tunable roll, the temperature of the SHC control terminal *T_c_* can be adjusted based on the stable temperature of the SHC reference terminal *T_r_*, and the dynamic adjustment of *T_c_* can be realized by adjusting the SHC control current. The time for reaching *T_c_* is generally within 60 s, which is far less than the control time of the profile-tunable roll. Therefore, it can be assumed that *T_c_* can reach the preset temperature at the initial state in the FE model of the profile-tunable roll. The boundary is driven by the controller set point $T_{c}$, and the analysis is interpreted in the steady or quasi-steady regime under the reported cooling; controller dynamics and short transients may introduce a brief timing lag that affects amplitude, but they do not change the spatial trend, which is the focus of this study. Therefore, *T_c_* is applied to one end of the silicone grease as the thermal boundary. The other end of the silicone grease is bonded to the roll, which can transfer the heat of the SHC control terminal to the roll. The specific parameters of the FE model are listed in Table 1.

**Figure 4:**
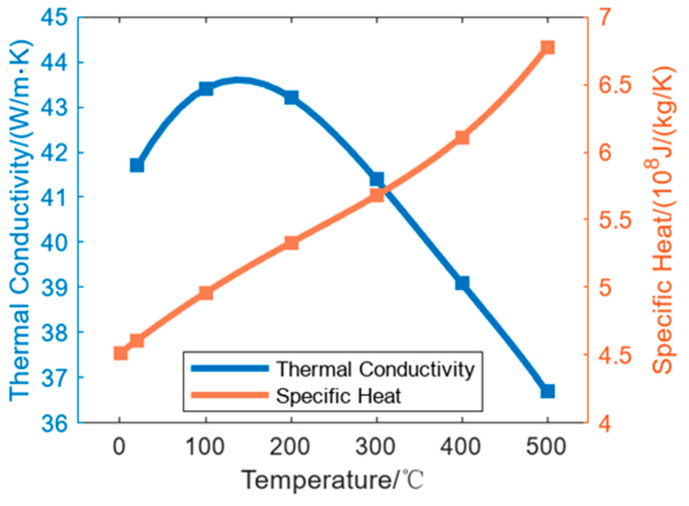
Thermal conductivity and specific heat of C45.

In the process of solid contact heat transfer, the heat transfer capacity is related to factors such as the pressure between two objects, the microscopic unevenness degree of the contact surface, and the effective contact area. Considering that the SHC and the roll are bonded with silicone grease, silicone grease has a certain viscosity and can effectively fill the area between the SHC and the roll. Therefore, the roll and silicone grease can be considered an adhesive contact. At the SHC-roll interface, we impose thermal contact conductance $h_{c}=2.4$ m^−2^K^−1^; the grease is not modeled as a separate meshed layer, and no equality to material thermal conductivity is assumed. The roll material is C45 with a density of 7.85 g/cm^3^, and the thermal property parameters are shown in Figure 4. The roll is cooled through air cooling, and its heat transfer coefficient is 5 W/(m^2^·K).

## 4. Heating Experiments of Profile-Tunable Roll

**Figure 5:**
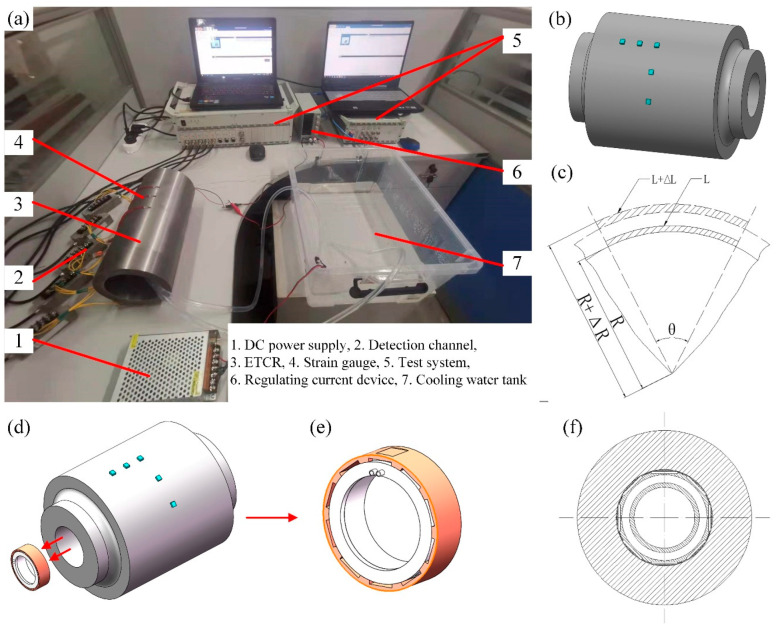
Experimental platform of profile-tunable roll and detection. (**a**) Experimental platform, (**b**) SHC layout, (**c**) schematic diagram of circumferential/radial elongation before and after bulging, (**d**) schematic diagram of the SHC ring assembled in the roll, (**e**) schematic diagram of the ring, and (**f**) cross-section drawing of the roll.

An experimental platform for the profile-tunable roll was constructed to measure the circumferential bulging under different operating conditions, as shown in Figure 5. The platform consists of a DC power supply, a detection channel, the profile-tunable roll, strain gauges, a data acquisition system, a current-regulating device, and a cooling-water tank. As illustrated in Figure 5b, strain gauges are mounted on the surface of the profile-tunable roll to measure the circumferential strain. Figure 5c shows the changes in the strain-gauge configuration before and after roll bulging. According to the geometric relationships, the configurations before and after deformation are expressed by Equations (5) and (6):

(5) Rθ=L

(6) $$\left(R+\Delta R\right)\theta =L+\Delta L$$

where *R* is the roll radius before bulging. Δ*R* is the radial bulging amount of the roll, and its value is equal to *ε_r_R*. *ε_r_* is the radial strain. *L* is the length of the strain gauge before bulging. Δ*L* is the stretching amount of the strain gauge, and its value is equal to *ε_c_L*. *ε_c_* is the radial strain. *θ* is the central angle corresponding to the length of the strain gauge.

Equations (5) and (6) are combined to obtain Equation (7):

(7) $$\varepsilon_{r}=\varepsilon_{c}$$

Therefore, the radial bulging of the roll can be obtained from the measured strain. SHC ring groups are assembled inside the roll, as shown in Figure 5d. The outer surface of each ring is fitted with multiple SHC modules distributed along the circumferential direction, whereas the inner surface contains a cooling-water channel through which the cooling water flows, as illustrated in Figure 5e. Silicone grease, highlighted in Figure 5e, is applied between the SHCs and the inner wall of the roll to ensure efficient heat transfer between them. In the experimental platform, the outer and inner diameters of the roll are 140 mm and 100 mm, respectively, and the roll length is 300 mm. Five SHCs were evenly installed along the circumferential direction of the roll. The experimental conditions are listed in Table 2. In this study, three repeats denote three independent heating–hold–cooling cycles, each initiated only after the system has returned to the same baseline state; summary statistics are computed across these independent cycles.

## 5. Results and Discussions

### 5.1. FE Model Verification

**Figure 6:**
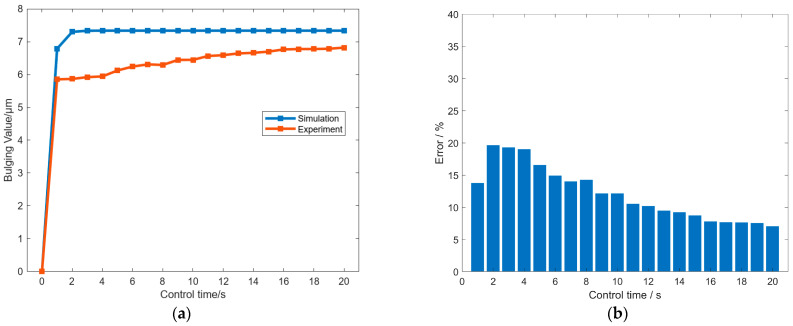
(**a**) Comparison of the maximum radial bulge from experiment and simulation. (**b**) Error between experiment and simulation results under different control times.

Figure 6a compares the maximum radial bulge of the roll obtained from the experiment and the simulation. The bulge increases rapidly during the initial control period and then approaches a stable value of approximately 7 µm. At early times, the experimental curve is slightly lower than the simulated one, with a maximum absolute deviation of about 1.5 µm; as the system approaches steady conditions, this deviation decreases to around 0.5 µm. The remaining discrepancy is mainly attributed to interfacial heat-transfer differences: In the FE model, the controller set point is applied at an ideal SHC-roll interface, whereas in the experiment, a thin paste layer and small gaps introduce additional thermal resistance and reduce the peak bulge amplitude. Overall, the two curves exhibit consistent trends in direction, temporal evolution, and magnitude. The experiment was repeated three times, each after returning to the same baseline, and yielded similar results, indicating good reproducibility. Based on these observations, the calibrated FE model is considered sufficiently accurate for analyzing the behavior of the profile-tunable roll under the reported conditions.

Prior thermal-crown and roll-profile control strategies primarily address axial or global crown with steady, quasi-uniform adjustments and provide little azimuthal addressability. Our method enables programmable circumferential control, supplying the missing θ-degree of freedom needed to mitigate azimuthal non-uniformities that limit R2P uniformity.

Figure 6b further quantifies the agreement between simulation and experiment at each control time. The relative error at time t is defined as the difference between the simulated and measured maximum radial bulges over the simulated maximum radial bulge. The bar chart shows a larger error during the initial regulation stage (about the first one to two minutes), followed by a steady decrease as the system approaches thermal equilibrium. Consistent with Figure 6a, the absolute difference in the early stage is within about 1.5 µm, which corresponds to a peak relative error on the order of tens of percent while the bulge is still growing. After the system reaches the quasi-steady regime (about 1200 s), the absolute difference falls to about 0.5 µm, and the relative error drops to a single-digit percent and remains at that level thereafter. This pattern indicates that the model captures the measured response within the stated error bounds, and the small discrepancy is mainly of the amplitude type rather than a change in circumferential pattern or stabilization times.

**Figure 7:**
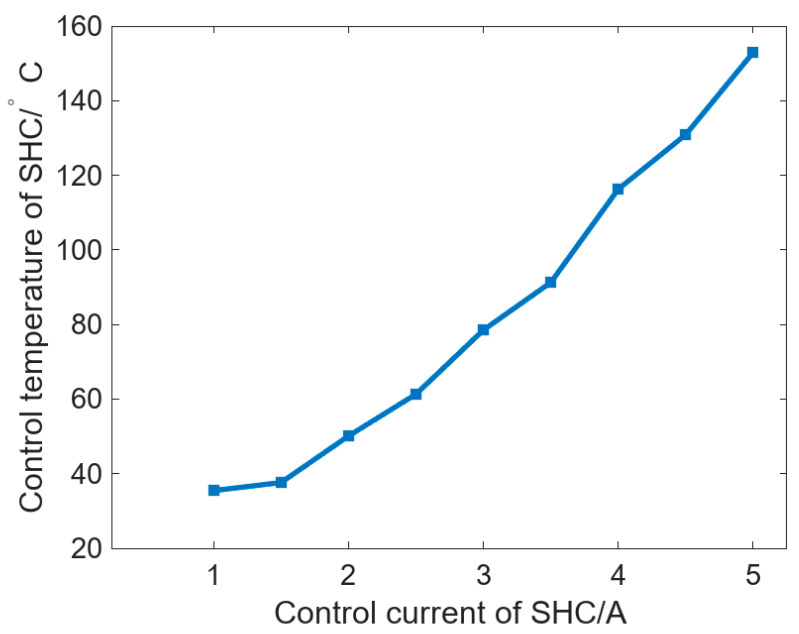
Variation of *T_c_* with the SHC control current when *T_r_* is 18 °C.

In addition, according to Figure 6a, the roll reaches quasi-steady conditions at approximately 1200 s, and the controller temperature $T_{c}$ has already stabilized before this. Figure 7, therefore, reports the variation in $T_{c}$ with the SHC control current at $T_{r}=$ 18 °C. The results show that currents of 1–5 A correspond to $T_{c}$ values of 35 °C, 50 °C, 78 °C, 116 °C, and 152 °C, indicating a near-linear increase in temperature with current. This circumferential control complements established axial crown methods to yield orthogonal 2D shaping (axial + azimuthal). Realizing a circumferential freeform pattern can be posed as an inverse problem in which the target surface figure is mapped to a required bulge distribution $C\left(\theta \right)$ and, via the transfer relation established here, to SHC set-points that generate the corresponding roll temperature field; end-to-end embossing and surface-profile verification are reserved for future work.

### 5.2. The Effect of Roll Size on Bulging Performance of Profile-Tunable Roll

To analyze the influence of SHC layouts and roll sizes on circumferential bulging, the maximum bulging difference is defined as the circumferential expansion difference (*C_ED_*), which is given by the difference between the maximum and minimum radial bulging of the roll, as expressed in Equation (8):

(8) $$C_{ED}=C_{\max}-C_{\min}$$

where *C_max_* is the maximum bulging, and *C_min_* is the minimum bulging.

**Figure 8:**
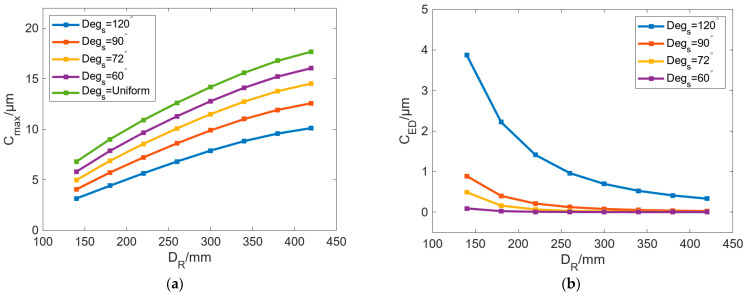
Variation in (**a**) *C_max_* and (**b**) *C_ED_* with increasing *D_R_* under different *Deg_s_*.

Figure 8 shows the variation in *C_max_* and *C_ED_* with *D_R_* under different *Deg_s_*. *T_c_* was set as 50 °C in these cases, and a uniform heat flow condition was also established as a reference. In the case of uniform heat flow, the element edges on the inner wall of the roll are set as the first thermal boundary with a value of 50 °C. In Figure 8a, *C_max_* can be increased with increasing *D_R_* and has a similar trend under different *Deg_s_*. With the increase in *D_R_*, the increasing rate of *C_max_* gradually decreases. After the *D_R_* reaches 420 mm, *C_max_* begins to stabilize and no longer increases as *D_R_* increases. As for the cases of different *Deg_s_*, when *Deg_s_* is decreased from 120° to 60°, *C_max_* can be gradually increased. Meanwhile, the influence value of *D_R_* on *C_max_* is 6.97 μm, 8.53 μm, 9.56 μm, and 10.3 μm, and the value is 10.88 μm in the uniform case.

In Figure 8b, *C_ED_* can be gradually decreased as *D_R_* increases. When *D_R_* is large enough, *C_ED_* is almost 0. We can see that when *D_R_* is small, the roll with uneven localized bulging is more conspicuous, and the larger the *Deg_s_*, the more obvious the unequal bulging in radial direction of profile-tunable roll. When *Deg_s_* is reduced from 120° to 60°, the maximum *C_ED_* values are 3.87 μm, 0.88 μm, 0.49 μm, and 0.09 μm. After *Deg_s_* is reduced to 90°, *C_ED_* is less than 1 μm.

**Figure 9:**
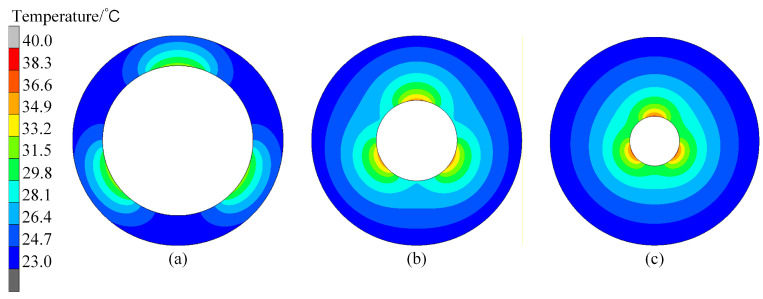
Roll internal temperature fields when *Deg_s_* is 120°. *D_R_* is (**a**) 140 mm, (**b**) 260 mm, and (**c**) 420 mm.

In the proposed profile-tunable roll, variations in the roll profile arise because the internal heat sources modify the temperature field, thereby inducing thermal bulging in different regions of the roll. According to the result in Figure 8, a conspicuous bulge exists in the case of *Deg_s_* = 120°. Therefore, the internal temperature field of the roll is illustrated in Figure 9. The results show that the temperature range above 28 °C can generate roll bulging, so 28 °C can be selected as the lowest temperature of the thermal bulging.

**Figure 10:**
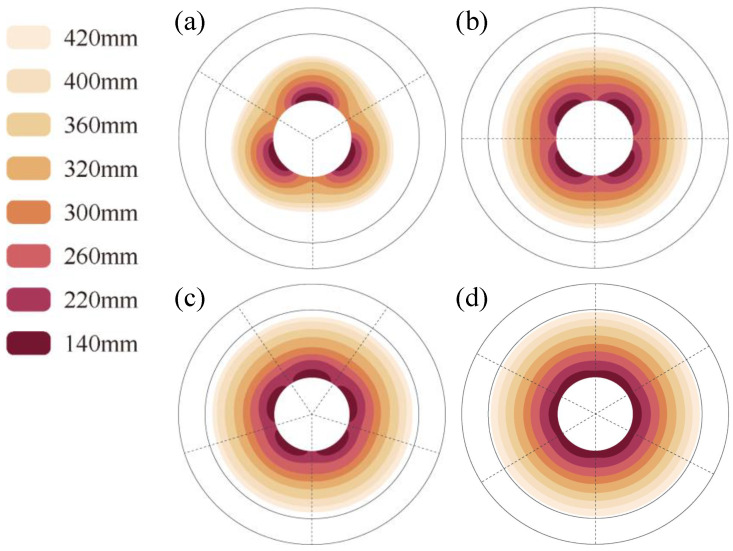
Variation in the 28 °C temperature-affected zone under different *D_R_*. *Deg_s_* values are (**a**) 120°, (**b**) 90°, (**c**) 72°, and (**d**) 60°.

Figure 10 shows the variation in the 28 °C temperature-affected zone inside the roll with *D_R_* under different *Deg_s_*. The results show that with increasing *Deg_s_*, the localized bulging is alleviated, which is consistent with the result in Figure 8b. With a smaller *D_R_*, reducing *Deg_s_* will lessen the localized thermal bulging caused by the SHC layout to a certain extent. When *Deg_s_* is 60°, the localized thermal bulging is even lower, which is nearly the same as uniform heat flow in the inner roll. Therefore, we can conclude that with a smaller roll diameter and larger *Deg_s_*, the temperature field with certain centrosymmetric bulging can basically be realized.

To further evaluate the effect of *Deg_s_* on temperature change, a new concept of Δ*T* was created to represent the regulating ability of temperature per unit time. It is defined as the ratio of the temperature difference to *Deg_s_*, and it can be calculated using Equation (9):

(9) $$\Delta T=\frac{T_{Deg\text{-}\max}-T_{Deg\text{-}\min}}{Deg_{s}}$$

where *T_Deg-max_* is the maximum temperature value within *Deg_s_*, and *T_Deg-min_* is the minimum temperature value within *Deg_s_*.

**Figure 11:**
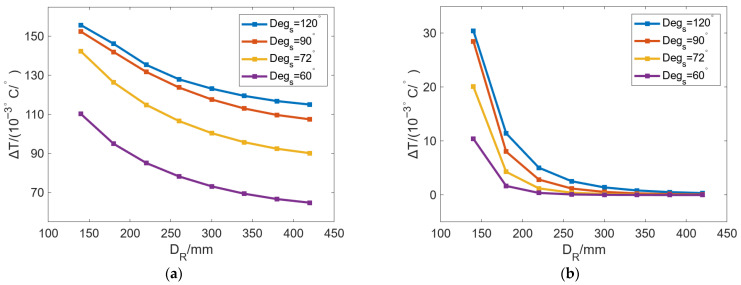
Variation in Δ*T* of the (**a**) inner and (**b**) outer walls of the roll with changing *D_R_* under different *Deg_s_*.

Figure 11 shows the change in Δ*T* of the inner wall and outer wall of the roll with *D_R_* changing under different *Deg_s_*. In Figure 11a, Δ*T* can be decreased as *D_R_* increases, with a gradually reduced decreasing rate. With the same *D_R_*, Δ*T* can be increased with increasing *Deg_s_*. When *Degs* is changed from 60° to 120°, Δ*T* can be gradually increased with increasing *D_R_*, which indicates that increasing *Deg_s_* can improve the localized bulging on the inner wall of the roll. In Figure 11b, the change in Δ*T* can be divided into two stages: The first stage is [140 mm, 220 mm]; Δ*T* can be decreased rapidly with increasing *D_R_*, and the rate of decrease slowly declines. The second stage is from 220 mm to 420 mm, where the decrease rate declines further and finally stabilizes, and with a continuous increase in *D_R_*, Δ*T* finally approaches 0 °C/°. Similarly, with the same *D_R_,* the increase in *Deg_s_* can lead to larger Δ*T*. When *D_R_* is 140 mm and *Deg_s_* decreases from 120° to 60°, Δ*T* can decrease from 0.03 °C/° to 0.01 °C/°. The larger the *Deg_s_*, the larger the drop. When *Deg_s_* is 120°, the maximum drop is 0.03 °C/°.

According to the above results, when *Deg_s_* exceeds 90°, the ratio of *β/α* is large, and the localized thermal bulging of the roll and the maximum difference of roll bulging are both large, which is suitable for optical plate forming with certain curved surfaces. When *Deg_s_* is less than 90°, the maximum difference value of roll bulging is small, and it will be further weakened by increasing *D_R_*, which is only suitable for freeform optical plates with extremely large curvature.

**Figure 12:**
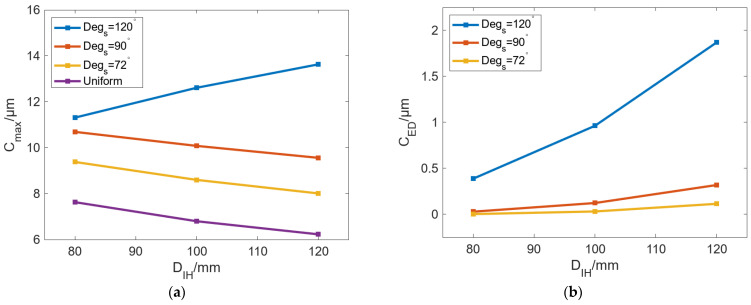
Variation in (**a**) *C_max_* and (**b**) *C_ED_* with increasing *D_IH_* under different *Deg_s_*.

In addition to the outer diameter of the roll, the diameter of the roll’s inner wall *D_IH_* is also an important parameter that can affect the bulging of the roll. When *D_R_* is 260 mm, moderate *C_max_* and small *C_ED_* can be achieved as the basic condition. To analyze the influence of *D_IH_* on circumferential bulging performance, *D_IH_* and *Deg_s_* have been investigated to analyze the bulging ability of the roll. Figure 12 shows the variation in *C_max_* and *C_ED_* with *D_IH_* under different *Deg_s_*. The results in Figure 12a show that with an increase in *D_IH_*, *C_max_* can be decreased, and the decrease rate of *C_max_* is the same under different *Deg_s_*. Under the same *D_IH_*, the increase in *Deg_s_* can reduce *C_max_*. When *Deg_s_* is increased from 72° to 120°, the change in C_max_ with increasing *D_IH_* is −1.13 μm, −1.37 μm, and −1.4 μm. On the whole, under the same *T*_c_, changes in *D_IH_* have a small influence on C_max_ with less than 2 μm. The results in Figure 12b show that *C_ED_* can be gradually increased with increasing *D_IH_*. When *Deg_s_* is 120°, the increased value of *C_ED_* is the largest with the value of 1.48 μm. When *Deg_s_* is 90° and 72°, the change values of *C_ED_* are 0.29 μm and 0.11 μm, respectively.

**Figure 13:**
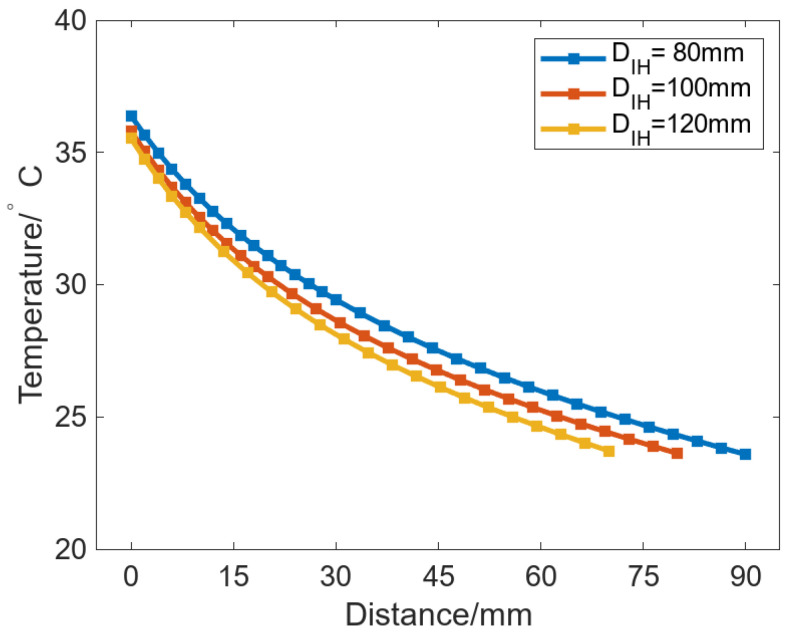
Node temperatures in the radial path from the roll inner wall to the maximum bulging point of the roll’s surface.

Moreover, Figure 12a also shows that the change trend of *C_max_* in the uniform case is different from that in other cases. *C_max_* can be gradually increased with increasing *D_IH_* in the uniform case, while *C_max_* can be decreased with increasing *D_IH_* in the other cases, where the case with *Deg_s_* of 120° is the most remarkable. To analyze the reason for this phenomenon, we extracted the node temperature when *Deg_s_* is 120°, which is located in the radial path from the inner wall of the roll to the maximum bulging point of the roll surface, as shown in Figure 13.

The results show that the larger the *D_IH_*, the lower the point temperature at the same distance from the inner wall of the roll, and thus, the corresponding thermal bulge at that position is smaller. This is because, for a constant outer roll diameter, increasing *D_IH_* is equivalent to reducing the roll wall’s thickness. As a result, the region near the roll surface can reach a higher temperature and form a larger thermal bulge. However, this effect exists only when there is no circumferential temperature difference or when the circumferential temperature difference is small. When the heat source is uniformly distributed in the circumferential direction, the bulging ability can be improved by decreasing the roll wall thickness. In the non-uniform case, the heat source is circumferentially non-uniform, so increasing *D_IH_* both reduces the roll wall’s thickness and increases the spacing between SHCs. For two adjacent SHCs, the symmetry plane between them also serves as the symmetry plane of the corresponding heat-transfer influence zones. With increasing *D_IH_*, the circumferential heat transfer between the adjacent SHC is also more obvious, which leads to a decrease in *C_max_*.

**Figure 14:**
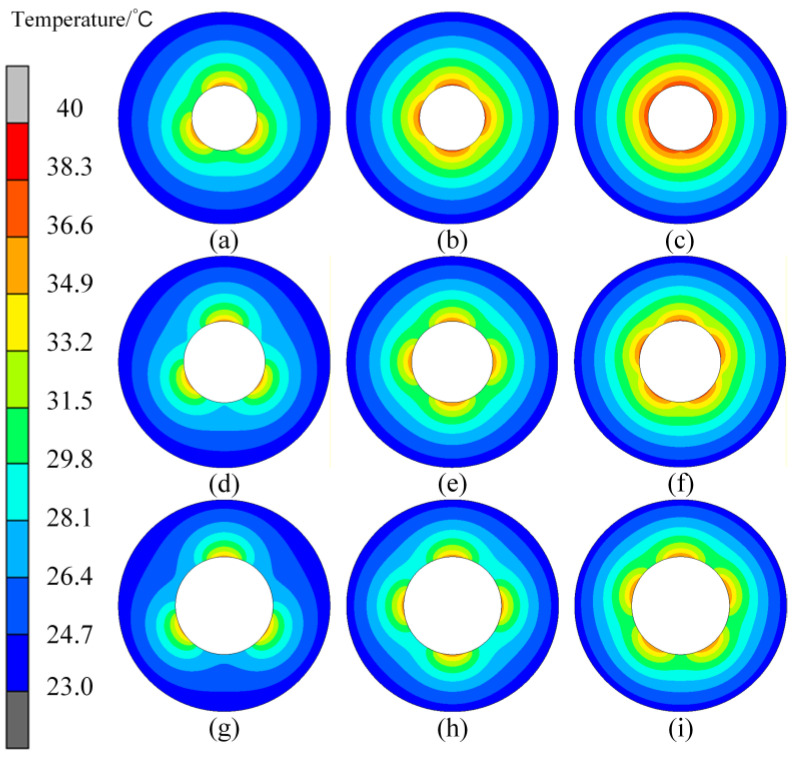
Variation in the roll temperature field under different *D_IH_* and *Deg_s_*. (**a**–**c**) *D_IH_* is 80 mm, and *Deg_s_* is from 120° to 72°; (**d**–**f**) *D_IH_* is 100 mm, and *Deg_s_* is from 120° to 72°; (**g**–**i**) *D_IH_* is 120 mm, and *Deg_s_* is from 120° to 72°.

Figure 14 shows the internal temperature fields of the roll with changing *D_IH_*. The results show that when *Deg_s_* is 120° in Figure 14a,d,g, there is an obvious difference in the internal temperature field of the roll regardless of whether *D_IH_* is large or small. When *Deg_s_* is reduced to 100°, the temperature difference is relieved, but when *D_IH_* becomes larger, such as Figure 14h, there will still be obvious thermal bulging. If *Deg_s_* is further reduced, thermal bulging in the case in which *D_IH_* is 120 mm will also be reduced.

The above changes occur because reducing *Deg_s_* increases the total direct influence angle, so no obvious thermal bulging appears under a uniformly applied heat flux. When *D_IH_* is increased, the total direct influence angle decreases, and the indirect influence angle increases, thereby reducing the uniformity of the heat flux on the inner wall of the roll and enhancing the thermal bulging.

**Figure 15:**
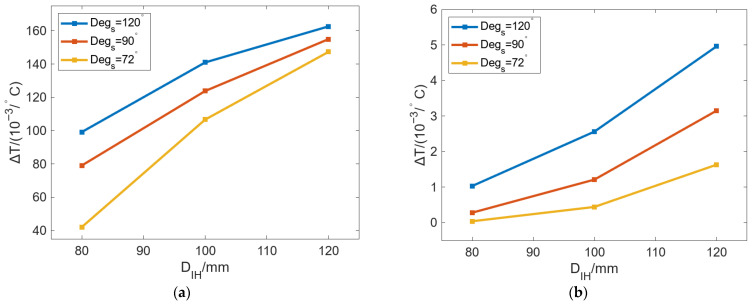
Variation in Δ*T* of the (**a**) inner and (**b**) outer walls of the roll with changing *D_IH_* under different *Deg_s_*.

Figure 15 shows the change in Δ*T* of the inner and outer walls with *D_IH_* under different *Deg_s_*. In Figure 15a, the Δ*T* of the inner wall of the roll can be increased with increasing *D_IH_*, but the growth rate gradually decreases. With the same *D_IH_*, a larger *Deg_s_* will result in a larger Δ*T*, and larger differences in the heat flow in the roll inner wall will occur. In Figure 15b, the Δ*T* of the outer wall of the roll can also be increased with increasing *D_IH_*, but the growth rate gradually increases. The difference in the growth rate of Δ*T* between Figure 15a,b is that increasing *D_IH_* can reduce the distance between the heat source and the roll surface, and the thermal bulging caused by different *Deg_s_* is more likely to appear on the outer wall of the roll.

Therefore, increasing *D_R_* can increase *C_max_* and decrease *C_ED_*, while increasing *D_IH_* can decrease *C_max_* and increase *C_ED_*. Changing *Deg_s_* can affect the effects of *D_R_* and *D_IH_*. Comparing the results of Figure 15b with Figure 12b, the cases in which *Deg_s_* is less than 90° have a relatively small maximum difference value of roll bulging than the cases in which *Deg_s_* is more than 90°, which is suitable for freeform optical plate forming with different curvatures.

### 5.3. The Effect of Temperature on Bulging Performance of Profile-Tunable Roll

**Figure 16:**
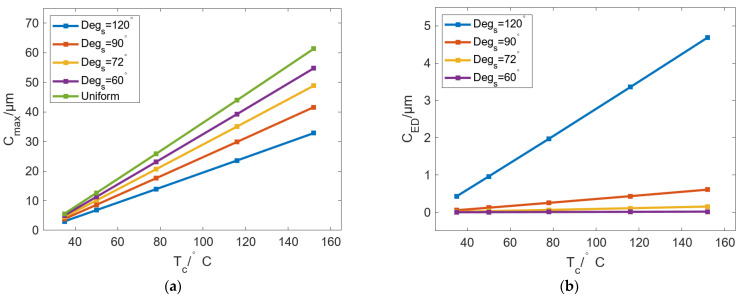
Variation in (**a**) *C_max_* and (**b**) *C_ED_* with increasing *T_c_* under different *Deg_s_*.

According to the above research, the bulging of the roll is relatively large, and *C_ED_* is small when *D_R_* is 260 mm and *D_IH_* is 100 mm. Therefore, these parameters are selected as basic conditions to analyze the influence of *T_c_* on thermal bulging. Figure 16 shows the variation in C_max_ and C_ED_ with *T_c_* under different *Deg_s_*. The results show that both *C_max_* and *C_ED_* can increase linearly with increasing *T_c_*. In Figure 16a, when *Deg_s_* is reduced from 120° to 60°, the growth rates of *C_max_* are 0.25 μm/℃, 0.32 μm/℃, 0.38 μm/℃, and 0.43 μm/℃. Compared with the uniform case of 0.48 μm/℃, the smaller the value of *Deg_s_*, the closer the bulging of the roll to the uniform case. In Figure 16b, in addition to the case in which *Deg_s_* is 120°, other cases have a lower *C_ED_*. When *Deg_s_* is decreased from 90° to 60°, the variation rates of *C_ED_* are 0.005 μm/℃, 0.001 μm/℃, and 0.0001 μm/℃. The results indicate that cases in which *Deg_s_* is 90°, 72°, and 60° have uniform circumferential bulging and cannot meet the requirements of radial roll shape with certain curves.

**Figure 17:**
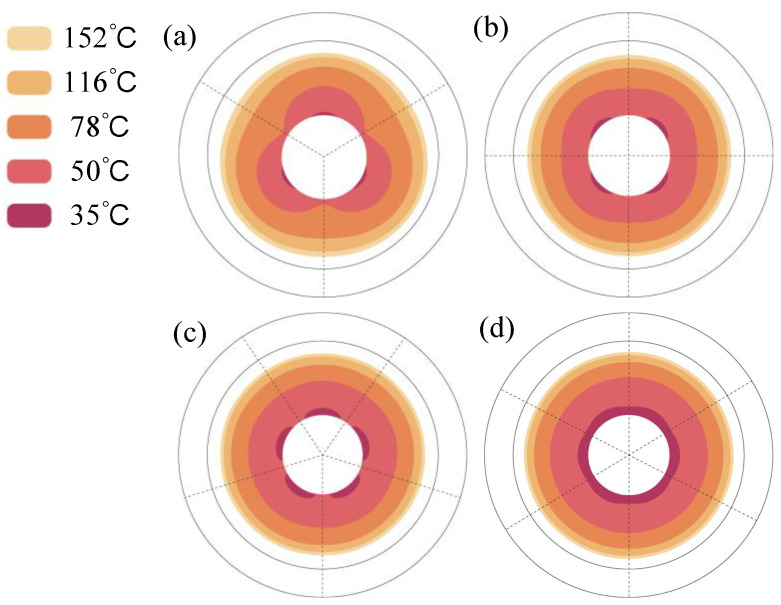
Variation in bulging in the zone where the temperature is above 28 °C, with *T_c_* under different *Deg_s_* of (**a**) 120°, (**b**) 90°, (**c**) 72°, and (**d**) 60°.

Figure 17 shows the bulging changes in the zone where the temperature is above 28 °C, with *T_c_* under different *Deg_s_*. The results show that when *Deg_s_* is 120°, only the case with a larger *T_c_* has a relatively small circumferential difference in the temperature field. In comparison, a case with a lower *T_c_* can achieve more obvious thermal bulging. Compared with the results in Figure 16b, the layouts with *Deg_s_* of 120° can be adopted in most cases of freeform plates with various curvatures in the R2P hot-embossing process regardless of whether *T_c_* is large or small. When *Deg_s_* equals 90°, 72°, and 60°, thermal bulging occurs only when the SHC control temperature is small, and thermal bulging will be reduced after slightly increasing *T_c_*.

**Figure 18:**
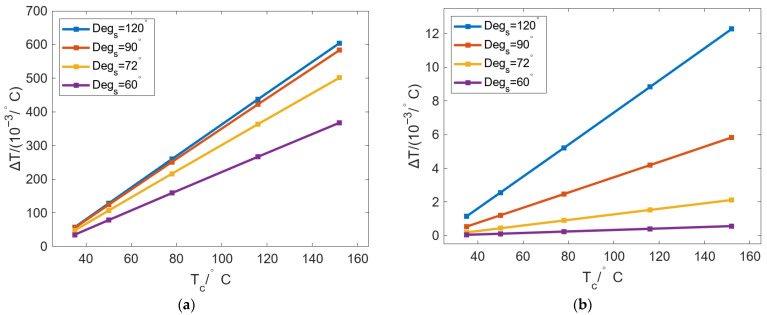
Variation in Δ*T* of the (**a**) inner and (**b**) outer walls of the roll with changing *T_c_* under different *Deg_s_*.

Figure 18 shows the change in Δ*T* of the inner and outer walls of the roll with *T_c_* under different *Deg_s_*. The results show that the Δ*T* of the outer wall and inner wall also increases linearly with increasing *T_c_*. In Figure 18a, the growth rate of Δ*T* increases with increasing *Deg_s_*, but the difference is not large. Specifically, the growth curves of *Deg_s_* 90° and 120° almost coincide in both value and trend, indicating that the heat flow distributions of the inner wall of the roll under these two cases are approximately the same. On the other hand, under the same *T_c_*, Δ*T* can be increased with increasing *Deg_s_*. In Figure 18b, the variation in Δ*T* with changing *T_c_* in the outer wall is much smaller than that in the inner wall. The changes of Δ*T* with *T_c_* and *Deg_s_* are also linear as in Figure 18a. Therefore, under the conditions of different *T_c_*, the temperature difference of the inner wall is relatively large and greatly affected by *T_c_*, while the temperature difference of the outer wall is very small. Hence, the increase in both *T_c_* and *Deg_s_* can lead to an increase in the circumferential temperature difference in the inner wall of the roll.

### 5.4. The Effect of SHC Number on Bulging Performance of Profile-Tunable Roll

**Figure 19:**
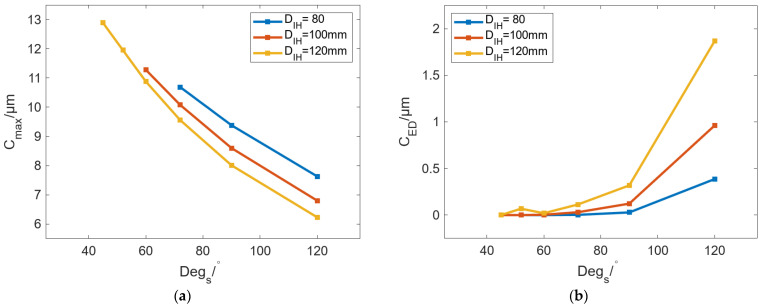
Variation in (**a**) *C_max_* and (**b**) *C_ED_* with *Deg_s_* under different *D_IH_*.

In addition to *T_c_*, the number of SHCs is also an important parameter in profile-tunable rolls. The circumferential SHC-carrying capacity of the roll is determined by the diameter of its inner bore. To investigate the relationship between the number of SHCs and the bulging performance, *D_R_* and *T_c_* are set to 260 mm and 50 °C, respectively, and *D_IH_* is set to 80, 100, and 120 mm. Figure 19 shows the variation in *C_max_* and *C_ED_* with *Deg_s_* under different *D_IH_*. The results show that under different *D_IH_*, *C_max_* can be decreased, and *C_ED_* can be increased with increasing *Deg_s_*. The change in *C_ED_* is divided into two stages: When *Deg_s_* is [40°, 90°], the difference in *C_ED_* under different *D_IH_* is small. It can be considered that changing *D_IH_* cannot affect the localized circumferential bulging achieved by *Deg_s_*. When *Deg_s_* exceeds 90°, the greater the *D_IH_*, the larger the localized circumferential bulging of the roll.

**Figure 20:**
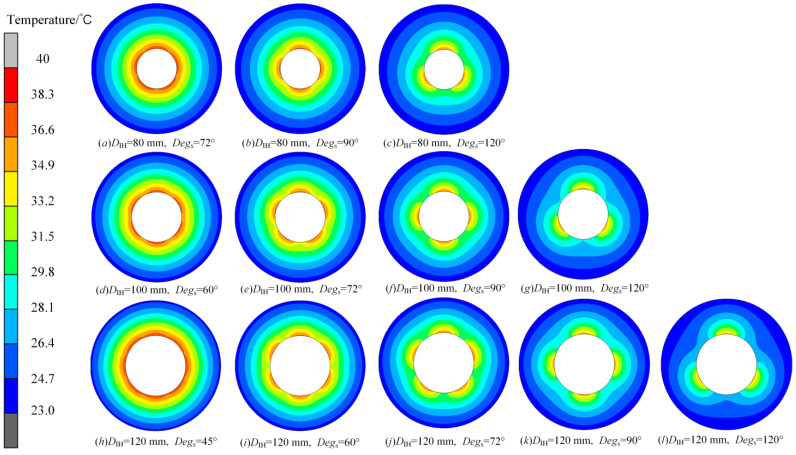
Variation in the roll temperature field under different *D_IH_* and *Deg_s_*. (**a**–**c**) *D_IH_* is 80 mm, and *Deg_s_* is from 72° to 120°; (**d**–**g**) *D_IH_* is 100 mm, and *Deg_s_* is from 60° to 120°; (**h**–**l**) *D_IH_* is 120 mm, and *Deg_s_* is from 45° to 120°.

Figure 20 shows the variation in the roll internal temperature field under different *D_IH_* and *Deg_s_*. The results show that, regardless of *D_IH_*, with an increase in *Deg_s_*, the circumferential thermal bulging can become increasingly obvious. In particular, when *Deg_s_* is 120°, the localized thermal bulging is so obvious that we can achieve three identical freeform optical plates when forming under this condition. Meanwhile, the curvature will be further reduced as *D_IH_* increases. It can be seen from the temperature distribution that the larger *D_IH_* is capable of generating more obvious localized bulging with fewer requirements for *Deg_s_*. For three conditions of different *D_IH_*, when *D_IH_* is 80 mm, *Deg_s_* should be no less than 90°; when *D_IH_* is 100 mm, the limitation for *Deg_s_* can be lowered to 72°; when *D_IH_* is 120 mm, any *Deg_s_* above 60° can generate micron-level bulging.

**Figure 21:**
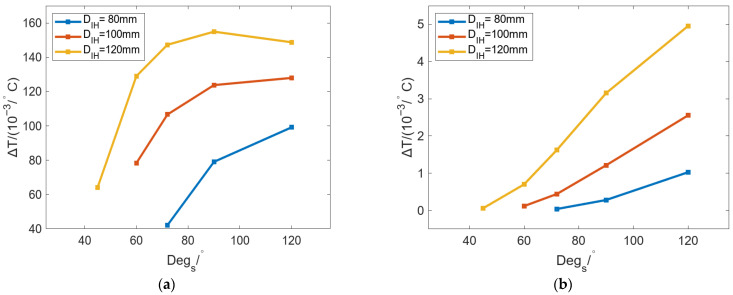
Variation in Δ*T* of the (**a**) inner and (**b**) outer walls of the roll with changing *Deg_s_* under different *D_IH_*.

Figure 21 shows the change in Δ*T* of the inner and outer walls of the roll with changing *Deg_s_* under different *D_IH_*. In Figure 21a, the curve of Δ*T* with *Deg_s_* was increased first and then gradually became stable. The smaller the *D_IH_*, the smaller the Δ*T* on the inner wall of the roll, and the heat flow of the inner wall will be more uniform. In Figure 21b, Δ*T* is also increased with *Deg_s_*, but it is much smaller than that in Figure 21a; this is to say that the Δ*T* of the outer wall of the roll is smaller, and the temperature distribution is more uniform.

### 5.5. Additional Copper Layer for Undesired Large Localized Bulging

**Figure 22:**
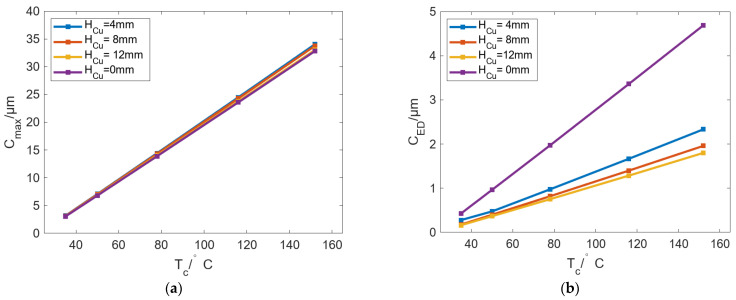
Variation in (**a**) *C_max_* and (**b**) *C_ED_* with increasing *T_c_* under different *H_Cu_*.

The above analysis shows that as *Deg_s_* increases, the number of localized bulges decreases while the bulging amplitude increases, making it difficult to achieve both a small number of localized bulges and small bulging values. To address this issue, a metal layer with high thermal conductivity can be arranged on the inner bore of the roll so that the heat supplied by the SHCs becomes more uniform along the inner surface, thereby reducing the bulging amplitude. In this study, a beryllium–copper layer with a thermal conductivity of 198 W/(m·K) is used as the metal layer on the inner bore of the roll, and the layer thickness *H_Cu_* is varied from 0 to 12 mm. Figure 22 shows the variation in *C_max_* and *C_ED_* with T_c_ under different *H_Cu_*. The results show that the increase in the beryllium copper layer has almost no effect on *C_max_*, but it can change the circumferential difference of the roll bulging. In Figure 22, the application of the beryllium copper layer can effectively reduce the *C_ED_* to 2 μm. If *H_Cu_* is further increased, *C_ED_* can be reduced by a small margin.

**Figure 23:**
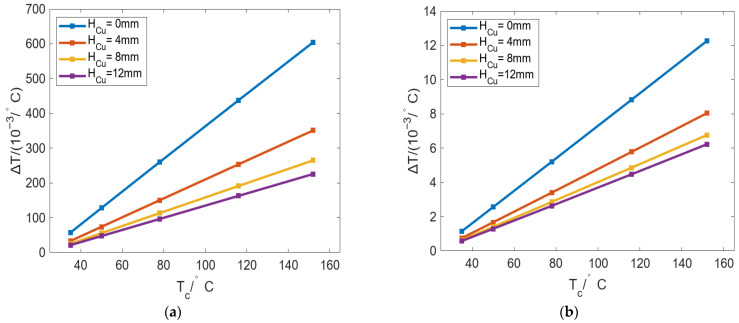
Variation in Δ*T* of the (**a**) inner and (**b**) outer walls of the roll with changing *T_c_* under different *H_Cu_*.

Figure 23 shows the change in Δ*T* of the inner and outer walls of the roll with changing *T_c_* under different *H_Cu_* values. In Figure 23a, Δ*T* can be decreased with increasing *H_Cu_*, indicating that the temperature near the roll inner wall is uniform. In Figure 23b, the influence of *H_Cu_* on Δ*T* is the same as that in Figure 23a, but the variation magnitude of Δ*T* is much smaller than that in Figure 23a. On the whole, by adding a thermally conductive metal layer to the roll inner wall, *C_ED_* can be reduced, and the bulging value for the cases with fewer bulging numbers can be alleviated.

## 6. Conclusions

In this paper, a novel R2P hot-embossing method for the fabrication of optical freeform plates was proposed by employing temperature-controlled, profile-tunable rolls. In the circumferential deformation simulations, the maximum circumferential bulging, the maximum bulging difference, and the roll temperature field were calculated and analyzed under different SHC numbers, SHC temperatures, and roll sizes. The main conclusions are as follows:

(1) The proposed profile-tunable roll was specially designed with a hollow structure to accommodate internal SHC modules. Variations in the outer and inner diameters, the single-piece influence angle, and the number of SHCs jointly exert a strong influence on circumferential profile deformation. Under different single-piece influence angles, increasing the outer diameter enhances the maximum bulging value from 3.12 µm to 17.64 µm while reducing the maximum bulging difference from 3.89 µm to nearly 0. In contrast, increasing the inner diameter decreases the maximum bulging value from 10.92 µm to 6.08 µm while increasing the maximum bulging difference from nearly 0 to 1.82 µm.
(2) The maximum bulging and temperature variation are also promoted by increasing the input temperature and decreasing the circumferential number of SHCs (i.e., increasing the single-piece influence angle). In addition, the single-piece influence angle has a much stronger effect on bulging deformation than the input temperature for different inner diameters. Micron-level bulging can be observed when the single-piece influence angles are no less than 90°, 72°, and 60° for inner roll diameters of 80 mm, 100 mm, and 120 mm, respectively.
(3) To relax the one-to-one correspondence between design parameters and both bulging amplitudes and the number of localized bulges, an internal circular copper layer was assembled on the inner bore of the roll. This layer effectively reduced the localized bulging amplitude to about 2 µm, and the amplitude can be further decreased by increasing the layer thickness.

## Figures and Tables

**Table 1:** Parameters of the FE model.

Parameter	Value	Parameter	Value
Roll diameter	140~420 mm	Hole diameter	80~120 mm
SHC size	40 mm × 40 mm	SHC thickness	5 mm
SHC control current	1–5 A	Silicone grease thickness	2 mm
SHC amount	3–5	SHC control time	1200 s
SHC control mode	Hot end control	Initial temperature	23 °C

**Table 2:** Experimental conditions of profile-tunable roll.

Ambient Temperature	Temperature of Cooling Water	Current	Time
20 °C	18 °C	3 A	1200 s

## Data Availability

The original contributions presented in this study are included in the article. Further inquiries can be directed to the corresponding author.
